# Effects of craving and DRD4 VNTR genotype on the relative value of alcohol: an initial human laboratory study

**DOI:** 10.1186/1744-9081-3-11

**Published:** 2007-02-19

**Authors:** James MacKillop, David P Menges, John E McGeary, Stephen A Lisman

**Affiliations:** 1 Department of Psychology, State University of New York at Binghamton, PO Box 6000, Binghamton, NY 13902-6000, USA; 2 Center for Alcohol and Addiction Studies, Brown University, Box G-BH, Providence RI 02906, USA; 3 Providence Veteran Affairs Medical Center, Providence RI 02909, USA

## Abstract

**Background:**

Craving for alcohol is a highly controversial subjective construct and may be clarified by Loewenstein's visceral theory, which emphasizes craving's behavioral effects on the relative value of alcohol. Based on the visceral theory, this study examined the effects of a craving induction on the relative value of alcohol as measured by a behavioral choice task. In addition, based on previous evidence of its role in the expression of craving, the influence of DRD4 VNTR genotype (DRD4-L vs. DRD4-S) was also examined.

**Methods:**

Thirty-five heavy drinkers (54% male; 31% DRD4-L) were randomly assigned to receive either a craving induction (exposure to personally relevant alcohol cues) or a control induction (exposure to neutral cues), which was followed by an alcohol-money choice task. Participants were assessed for craving and positive/negative affect throughout the procedure, and relative value of alcohol was derived from participant choices for alcohol versus money. DRD4 VNTR status was assessed retrospectively via buccal samples using previously established protocols.

**Results:**

Factorial analysis of the craving induction revealed that it was associated with significant increase in craving (*p *< .001), but not greater relative value of alcohol. Factorial analyses including DRD4 VNTR genotype of did not suggest an influence on reactivity to the craving induction, although this analysis was substantially compromised by small cell sample sizes. Continuous analyses revealed that craving was significantly associated with the relative value of alcohol (*p *< .05) and possession of the DRD4-L allele further amplified this relationship (*p *< .001).

**Conclusion:**

These results are interpreted as generally supporting Loewenstein's visceral theory of craving and evidence of a functional role of DRD4 VNTR genotype in the expression of craving for alcohol. Methodological limitations, mechanisms underlying these findings, and future directions are discussed.

## Background

Despite the long history of research on craving for alcohol [e.g., [1]], the functional relationship between craving and both alcohol use and post-treatment relapse remains highly contentious [2,3]. This controversy is, in part, a result of considerable ambiguity in the data connecting craving and alcohol misuse. Although alcohol dependent individuals readily report experiencing cravings for alcohol [4,5], the associations between self-reported craving and actual alcohol use in human laboratory studies have been equivocal [for a review, see [6]; cf. [7,8]]. Similarly, in clinical research, a number of retrospective studies have reported a negligible role of craving in relapse [9-11]. Given the ambiguous empirical data, it is not surprising that there is little consensus as to the role of craving in alcohol use disorders [2,12].

The present study sought to better understand craving for alcohol using a novel theoretical approach, Loewenstein's [13,14] visceral theory of addiction. The visceral theory is a hybrid theory that integrates decision theory [e.g., [15,16]] and learning theory [e.g., [17,18]] via a common pathway of behavioral economics. From this perspective, addiction is an extreme example of a wide array of behaviors that are largely controlled by visceral factors, or drive states such as hunger, thirst, sexual desire, or, in the case of addiction, craving. The decision to use a substance is considered to be the final determinant of substance use, which is itself guided by the value of the substance relative to all other available choices (i.e., relative value). However, the relative value of a substance is not a static property and it is proposed to be substantially influenced by a number of variables, most prominently, craving. Specifically, the visceral theory proposes that it is not simply the experience of craving that precipitates substance use, but rather it is the *effects *of craving, including increased relative value of the substance, increased discounting of delayed rewards, and narrowed temporal perception, among others. Over time, substance misuse is proposed to be a function of these effects recurrently biasing an individual's behavior toward continued substance use over more salubrious options. Importantly, by virtue of its emphasis on craving's effects on the value of the substance, Loewenstein's visceral theory is distinct from other contemporary theories of craving that emphasize craving as a classically conditioned response [18-20], an affective process [21,22], or a cognitive process [23]. In addition, the visceral theory can be clearly distinguished from approaches that propose craving to primarily reflect the interruption of automatized addictive behaviour, and, as a result, to be largely epiphenomenological [6,24].

Although the visceral theory has not been subjected to substantial direct empirical testing, a number of its predictions have been borne out by the empirical literature [e.g., [25-27]]. This includes one of the visceral theory's central predictions, that craving increases the relative value of a substance, which has received provisional empirical support in a number of studies. These studies have largely been in reference to tobacco dependence, where evidence that craving can be understood in measures of economic value has been obliquely supported by several laboratory studies using craving-related manipulations (e.g., enforced abstinence) that resulted in increases in the relative value of smoking [28-30]. However, because these studies were not intended to directly test the visceral account, the majority lack critical variables, such as the subjects' level of craving, or did not report the relationship between craving and the relative value of smoking.

More convincingly, three studies have directly examined and reported the relationship between craving and the relative value of smoking with generally positive results. In an initial study, Willner et al. [31] found a significant increase in the relative value of tobacco following deprivation using a progressive-ratio operant task. Similarly, Perkins et al. [32] found a positive association between craving and subjects' perceived cash value of tobacco, although craving was not associated with effort expended on an operant task. Finally, Sayette et al. [33] conducted a multi-dimensional assessment of cue-elicited craving and found a significant association between craving and the relative value of smoking as measured using a forced-choice self-administration task.

Comparatively little research from this perspective has been conducted in reference to craving for alcohol, with only one study in that domain. In the context of a laboratory study on the mechanisms of naltrexone pharmacotherapy, O'Malley et al. [7] administered a priming dose of alcohol and then used a self-administration paradigm that permitted participants to "buy" drinks. Post-priming subjective craving was found to significantly correlate with the number of alcoholic beverages chosen (and money foregone), thus converging with the majority of evidence from tobacco research that subjective craving can be understood in terms of relative value. Considered together, the preceding studies on craving for tobacco and alcohol suggest that the visceral theory is a promising approach for understanding craving. To further test the theory, the first goal of the current study was to examine the effects of a craving induction on the relative value of alcohol.

The second goal of the current study sought to examine a potential neurogenetic variable that may influence the relationship between craving and the relative value of alcohol. There is considerable evidence that one of the major neural substrates of craving is mesolimbic dopaminergic neurotransmission [34,35], although not exclusively [e.g., [36]]. Moreover, there is recent evidence that alleles of the gene responsible for the dopamine D_4 _receptor, which is localized within the limbic system, may play an important role in the expression of craving for alcohol [37-40]. Specifically, the D_4 _receptor gene (DRD4) has a variable number of tandem repeats (VNTR) polymorphism in exon 3, with common variants of 2, 4, and 7 repeats [41], and there is evidence that possession of a long allele (7 repeats or more; DRD4-L) confers functional differences in dopamine neurotransmission [42-44]. Vis-à-vis craving, previous studies have demonstrated that DRD4-L status positively moderates the effects of both an alcohol cue exposure [37,40] and priming dose of alcohol on craving [38]. Similar interactions between DRD4 VNTR genotype and craving have also been found in laboratory studies of smoking [45], heroin addiction [46], and binge eating [47]. Although the exact role of the DRD4 VNTR genotype remains unclear, these studies suggest that possession of a long version of the DRD4 VNTR genotype may also enhance the effect of craving on the relative value of alcohol.

Thus, this study was a preliminary investigation using the visceral theory as a framework for concurrently examining the influences of a craving induction and DRD4 VNTR genotype on the relative value of alcohol. Heavy drinkers were enrolled in a laboratory protocol and randomized to receive either a craving induction (i.e., exposure to personally relevant alcohol cues) or a control induction (i.e., exposure to neutral cues), followed by an alcohol-money choice task to assess the relative value of alcohol. A cue exposure approach was employed as the craving induction because it has been robustly validated for inducing craving in the laboratory [for a review, see [48]], and has the advantage of not including the array of concomitant physiological effects of induced withdrawal [e.g., [30]] or mild intoxication [7]. Moreover, the visceral theory proposes that the most important form of craving is cue-elicited craving that persists beyond treatment [13,14]. Based on the visceral theory, the craving induction was predicted to be associated with greater relative value of alcohol compared to the control induction, and the absolute level of craving independent of induction was predicted to be significantly associated with relative value of alcohol. Although the existing literature on DRD4 VNTR effects is relatively small, DRD4-L status was predicted to both positively moderate the effects of the craving induction, replicating two previous studies [37,40], and the continuous relationship between craving and the relative value of alcohol. Based on theoretical interest in the relationship between craving and affect [21,22,49], positive and negative affect was concurrently assessed throughout the study, but given ambiguous associations in previous studies [e.g., [49]], no directional predictions were made.

## Methods

### Participants

Participants were recruited using posted advertisements and classroom/email solicitations at the State University of New York at Binghamton. They were required to be heavy drinkers, defined as drinking at least 20+/14+ standard drinks per week for males/females [50] and of legal age for alcohol consumption (21 years old in New York State). Since the multimodal craving induction was oriented around beer, prospective participants were required to report beer as both a favorite and most often consumed alcoholic beverage, and to rate their enjoyment of beer seven or greater on a 10-point Likert-type scale. Participants were compensated with $10, plus any money selected during the choice task.

Thirty five (54% male; mean age = 21.83 [*SD *= 1.12]) heavy drinkers were enrolled in the study. Male participants drank an average of 36.32 (*SD *= 12.98) drinks/week and female participants drank an average of 21.00 drinks/week (*SD *= 6.54). These levels reflect the 97^th ^and 98^th ^percentiles of alcohol consumption for this cohort [51]. The mean AUDIT score was 14.2 (*SD *= 5.58) and 100% of participants scored an 8 or higher, the previously validated criterion for hazardous drinking [52]. Eleven (31%) participants were classified as DRD4-L based on possession of at least one version of the DRD4 VNTR allele with seven or more repeats. Median household income was $100,000 (IQR = $65,000–$135,000). In terms of race/ethnicity, 83% of the sample was Caucasian, 14% of the sample was Hispanic, and 3% of the sample was Asian.

### Experimental design

The study used a one-way two-group between-subjects design. Upon enrollment, participants were randomly assigned to receive either the craving induction or control induction and all underwent the relative value of alcohol task immediately following the post-induction assessment. In considering DRD4 VNTR influences, the design was elaborated to 2 (craving induction/control induction) × 2 (DRD4-L/DRD4-S) quasi-experimental factorial design. The latter was quasi-experimental because subjects cannot be randomly assigned to genotype. In addition to the factorial design, based on the visceral theory's emphasis on the importance of the absolute level of craving [13,14], continuous relationships were also examined and are described further in the Data Analysis section.

### Measures

#### Drinking days questionnaire (DDQ)

The DDQ is a seven-item, face-valid measure of an individual's average alcohol consumption per week. Its seven items assess the typical amount consumed for each day of the week and it has been shown to have adequate psychometric properties [53,54].

#### Alcohol use disorders identification test (AUDIT)

The AUDIT [52] is a ten-item self-report screening measure that evaluates various quantitative and symptom-related aspects of drinking. The AUDIT yields a score from 0–40; a score of 8 or above indicates hazardous drinking, or drinking at risk for negative psychosocial consequences. The AUDIT has been extensively validated in terms of psychometric properties with high sensitivity and specificity [52], and had a coefficient α of .74 in this sample.

#### Subjective craving

A single item measure of craving [e.g., [33,35]] was employed in this study and followed recommendations for conceptualizing craving on a continuum of urges for alcohol [56,57]. In this case, a 100-point Likert-type scale of urge to drink was used with four anchoring comments (*"I don't want a beer at all," "I don't want a beer very much," "I'd like a beer now," *and *"I'd REALLY like a beer right now!!!"*) to enhance the likelihood of similar interpretation of the scale by participants. The first and last comments were placed at the low and high ends of the scale, respectively, and the two intermediate comments were beneath the numbers 35 and 70, respectively.

#### Positive and negative affect schedule (PANAS)

Positive and negative affect were assessed using the PANAS [58]. The PANAS is a 20-item measure of transient mood that has undergone extensive previous psychometric validation [58,59]. In this sample, under neutral conditions, the positive affect subscale of the PANAS generated a baseline coefficient α of .87 and the negative affect subscale generated a baseline coefficient α of .85.

#### Relative value of alcohol task

The relative value of alcohol task was based on previous methods used to assess relative value in behavioral economic research [29,60,61]. Because craving states are relatively transient [62,63] and delays of rewards substantially influence their relative value [60], the task was designed to be as short as possible (~90 seconds) and all alcohol and/or money selected was provided immediately following the task. To maximize the validity of the choice task, participants were provided with actual alcohol and/or money based on their choices.

Procedurally, each participant's relative value of alcohol was empirically defined in monetary terms using a series of choices to determine the average amount of money a participant would "pay" for a sip of alcohol. Participants completed nine choices between a 1.5-ounce sip of the participant's favorite beer (alcohol) and varying amounts of money to determine this point. This sip volume was selected because the total volume of alcohol available was one standard beer and 1.5 ozs represented approximately one ninth of the beer, which was an appropriate size for a sip. In addition, this unit was easily conveyable to participants. The first choice was between a sip of alcohol and $1, and the overall domain of potential choices and increments was as follows: $.01, $.02, $.05, $.10, $.25, $.50, $1 (starting point), $1.50, $2, $2.50, $3, $3.50, $4, $4.50, and $5.

The task used an adaptive adjusting procedure based on the participant's responses over the course of the nine choices. If the participant chose alcohol, the amount of money offered on the next choice was increased by one increment; if the participant chose money, the amount of money offered on the next trial decreased by one increment. Relative value of alcohol was defined as the value that reflected the individual's change in preference from alcohol to money and vice versa [i.e., "crossover" point; [29]]. Specifically, relative value of alcohol was defined as the mean of the smallest amount of money chosen over a sip of alcohol (inferring that alcohol was worth less than that amount) and the largest amount of money over which alcohol was chosen (inferring that alcohol was worth more than that amount). Of note, the critical dependent variable in this study was relative value of alcohol, not the absolute amount of money earned in the task. As a result of the adjusting procedure in the task, there was not a linear relationship between the number of choices made for money and the amount of money earned. Equally, since the adaptive adjusting procedure was responsive to choice, the absolute numbers of choices for money or alcohol were redundant with relative value of alcohol and not used as dependent variables.

### Genotyping

A sample of buccal cells was collected from each participant using an oral swab, following previously published procedures [37]. Participants swabbed their cheeks with three cotton swabs and then rinsed their mouths with 10 ml distilled water. The swabs and wash were placed in a sterile 50 ml polypropylene tube containing 500 μl of 1 M Tris-EDTA buffer (1 M Tris-HCl, 200 μM disodium EDTA, pH 8.0), 500 μl of 5% sodium dodecyl sulfate (SDS), and 100 μl of 5 M sodium chloride. The tubes were refrigerated until DNA was extracted. To extract the DNA, proteinase K (0.2 mg/ml) was added to the tubes and the tubes were incubated at 65°C for 60 min. The swabs were removed and residual lysis buffer was extracted by centrifugation (using a 3-ml syringe barrel and sterile 15 ml tube) for 5 minutes at 1,000 × g. The residual fluid was added back to the original sample. An equal volume of isopropyl alcohol was then added to each tube, the contents were mixed, and the DNA was collected by centrifugation at 3,500 × g for 10 min. The DNA pellet was rinsed once with 1 ml of 50% isopropyl alcohol and allowed to air dry. The pellet was resuspended in 1 ml of 10 μM TRIS-HCl, 10 mM EDTA buffer (pH 8.0) and place in a 1.8-ml cryovial. The concentration of DNA was calculated from the absorbance at 260 nm. Samples were stored at -70°C until used. The collection and extraction rate was 100%; all participants were retrospectively genotyped.

The 48 basepair VNTR in exon 3 of the DRD4 gene was assayed using primer sequences: forward, 5'-AGGACCCTCATGGCCT TG-3' (fluorescently labeled), and reverse 5'-CGACTACGTGGTCTACTCG-3' [64]. Genotyping success rate was 100%. Consistent with previous research [37-40], participants who were homozygous or heterozygous for an allele of seven repeats or longer were classified as DRD4-L and all other participants were classified as DRD4-S. The allele frequencies of the sample are presented in Table 1.

**Table 1:** DRD4 VNTR allele and genotype frequencies; percentages do not always add to 100 due to rounding error.

Allele/genotype	*n*	%
Allele		
2	8	11.4
3	3	4.3
4	46	65.7
5	1	1.4
6	0	0
7	11	15.7
8	0	0
9	1	1.4
Total	70	99.5
Genotype		
2-2	1	2.9
4-2	4	11.4
7-2	2	5.7
4-3	2	5.7
7-3	1	2.9
4-4	16	45.7
5-4	1	2.9
7-4	6	17.1
9-4	1	2.9
7-7	1	2.9
Total	35	100.1
Genotype Classification		
DRD4-L	11	31.4
DRD4-S	24	68.6
Total	35	100

DRD4-L subjects have at least one allele that is 7 repeats or longer; DRD4-S participants have both alleles shorter than 7 repeats.

### Procedure

All study procedures were approved by the Human Subjects Research Review Committee at the State University of New York at Binghamton. The experiment involved one laboratory session that took place in the afternoon and lasted 90-minutes. Participants were informed that the session would last the full duration to avoid any perceived contingency between not drinking and early departure, and that they would be compensated $10 but could also potentially receive additional money. In addition, participants were informed that they would have access to alcohol [65] and would be required to endorse that there were no significant reasons why they could not drink if they chose to during the session (e.g., medication, athletic event, examination). In advance of the experimental session, participants were sequentially randomized to receive the craving or control induction.

During the experimental session, participants initially provided informed consent in a neutral experimental room and were assessed for breath alcohol. If any breath alcohol was detected, the session would be terminated and they would be rescheduled; no participants required rescheduling. Participants completed the alcohol use, craving, and affect measures. They were then escorted to either the alcohol or neutral cue exposure room and underwent the craving induction or control induction.

The craving induction consisted of exposure to an array of personally relevant alcohol cues, including visual, olfactory, tactile, imaginal, and proprioceptive cues. Participants were introduced into a dimmed laboratory room (8 ft × 6 ft × 8 ft [2.44 m × 1.83 m × 2.44 m]) decorated with beer-related paraphernalia (posters and advertisements of beer, barroom trifolds, empty beer bottles). A Research Assistant (RA) then brought a bottle of the participant's favorite beer and poured it into a beer glass. Participants were then left alone for 60 seconds to observe the array of cues. After one minute, the RA returned and informed the participants that they would be asked to listen to an imaginal scene. In addition, participants were told that during the scene they would be asked to smell the beer from time to time. To establish the correct behavior, participants were then asked to lift the beer to their nose and take five inhalations of the smell of the beer. The RA then provided instructions for listening to the scene and left the room, observing via a one-way window that the participant correctly followed the scene-related instructions. Over the course of the scene, participants were asked to hold the beer up to their nose and deeply inhale the smell of the beer for five seconds on five occasions (total olfactory exposure with practice = 30 seconds). The imaginal scenes were developed based on previous research on imaginal scenes in cue reactivity research [66,67] and described common environmental, interpersonal, and affective contexts of drinking, as well as evocative descriptions of the orosensory properties of beer. Participants were matched to their favorite brand of beer [63] and were also matched to the imaginal scenes that related to their most common reason for drinking from among seven possibilities: relaxation, happiness, enjoyment of the taste, anger, boredom, sadness, anxiety, or habit. Favorite beer and appropriate imaginal scene had been assessed during screening; 32 participants received the happy scene, 2 received the bored scene, and 1 received the sad scene. The control induction was matched in each respect but took place in a different room and all cues, including imaginal scene, were oriented around consuming water. For both groups, the cue exposure periods were equivalent (~8 min).

Following the cue exposure, participants completed the craving and affect measures a second time, and then completed the relative value of alcohol task. Participants were informed that they now had the opportunity to choose either a 1.5 oz sip of their favorite beer or various amounts of money. The volume of the sip was demonstrated using water in a glass. In addition, the participants were informed that as soon as the task was over, they would be provided with the total amount of alcohol and/or money that they chose. The provision of the alcohol/money concluded the experimental portion of the procedure. Participants then returned to an undecorated experimental room (without either alcohol or water cues) and underwent the genotyping procedure, before relaxing in that room for the remainder of the session (approximately one hour). This period was used to ensure that no participants would leave the study intoxicated. At the conclusion of the session, a debriefing was conducted and breath alcohol was established at 0.00%. All genotyping was conducted retrospectively, following the completion of enrollment.

### Data analysis

All baseline data were examined for outlying data points and distribution normality. All participants were examined on alcohol use and other self-report variables to evaluate potential baseline differences as a result of induction assignment or DRD4 VNTR genotype. Principal analyses used hierarchical multiple regression to examine the influence of covariates, induction type, genotype, and induction-by-genotype interaction; the latter term was examined for evidence of a moderating genetic influence [68]. Following the factorial analyses, continuous analyses were conducted between the experiential variables and the relative value of alcohol. The difference between the factorial and continuous analyses was that the former used the induction type (i.e., craving versus control induction) as an independent variable, whereas the latter used the participants' actual values for the experiential variables (i.e., craving and affect) as an independent variable. Both factorial and continuous approaches were employed because Loewenstein's [13,14] visceral theory proposes that the absolute level of craving is the most significant determinant of the relative value of alcohol and a continuous analysis addressed this more directly. Specific analytic strategies for each portion of the study are provided in the Results section. In all cases, statistical significance was determined by examining the initial regression model fit (*R*^2^, *F*-ratio) and relative improvement of the model fit (Δ*R*^2^, *F*-ratio) in subsequent blocks. Where multiple variables were included in a multiple regression block, each variable was evaluated by examining the significance of the variable coefficient. Follow-up analyses were conducted to examine the possibility of population stratification [69], a "third variable" confound based on differential associations between a given phenotype and racial/ethnic differences. For all significant genetic findings, population stratification was addressed by re-examining the findings within the largest single racial group (a group with minimized racial admixture).

## Results

### Baseline analyses

All baseline dependent variables were adequately normally distributed. No data were missing and no outliers were identified. Using one-way analyses of variance (ANOVAs), no significant baseline differences were evident between participants randomized to the two inductions on the following variables: drinks/week, AUDIT score, craving, positive affect, and negative affect (all *p*s > .20). One-way ANOVAs also revealed no significant differences between DRD4-L and DRD4-S genotypes on the following variables: drinks/week, AUDIT score, craving, positive affect, and negative affect (all *p*s > .20). Because of the potential relevance of income, drinks/week, and level of problems with alcohol (i.e., AUDIT score) to the dependent variables, these variables were also initially examined as potential covariates using simple regressions. Coefficient estimates revealed that income, drinks/week, and AUDIT were not significantly associated with post-induction craving (*p*s > .10), relative value of alcohol (*ps *> .20), and positive affect (*p*s > .20). However, drinks/week was significantly negatively associated with post-cue exposure negative affect (β = -.52, *p *< .01) and was included as a covariate in the subsequent negative affect analyses; income and AUDIT score were nonsignificantly associated with post-induction negative affect (*p*s > .14).

### Influences of the craving induction and subjective craving on the relative value of alcohol

**Figure 1:**
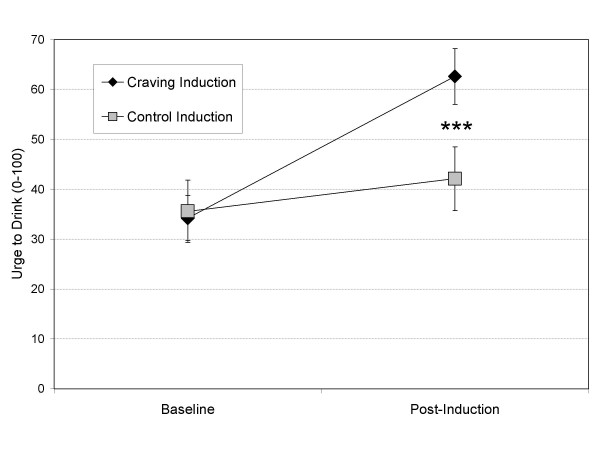
Effects of the craving induction (personalized alcohol cues) and control induction (neutral cues) on urge (i.e., craving) for alcohol; *** *p *< .001.

Induction effects were examined using hierarchical multiple regression to sequentially examine the influence of covariates and the effects of the experimental manipulation, with the post-induction values of a variable serving as the dependent variable, its pre-induction level entered as a covariate in a first block (with the exception of relative value of alcohol, which had no baseline level), and induction type entered into a second block. For the effects of the craving induction, the covariate model was significant (*R*^2 ^= .54; *F *(1, 33) = 38.35, *p *< .001), indicating that baseline craving was significantly associated with post-induction craving. The addition of the induction type in a second model significantly improved the model (Δ*R*^2 ^= .17, *F *(1, 32) = 18.52, *p *< .001) and reflected a significant increase in craving for individuals receiving the craving induction compared to the control induction, as depicted in Figure 1. For positive affect, the covariate model was significant (*R*^2 ^= .88, *F *(1, 33) = 261.79, *p *< .001), but the addition of induction type did not significantly improve the model (Δ*R*^2 ^= .00, *F *(1, 32) = .05, *p *> .80). For negative affect, the covariate model including both drinks/week and negative affect was significant (*R*^2 ^= .77, *F *(2, 32) = 54.80, *p *< .001), but the addition of induction type did not significantly improve the model (Δ*R*^2 ^= .00, *F *(1, 31) = .23, *p *> .60). Coefficient estimates for the model revealed a significant association with baseline negative affect (*p *< .001) and a marginally significant association of drinks/week (*p *= .10). In terms of the effect of the craving induction on the relative value of alcohol, no significant effect was evident (*R*^2 ^= .01, *F *(1, 33) = .02, *p *> .80).

**Figure 2:**
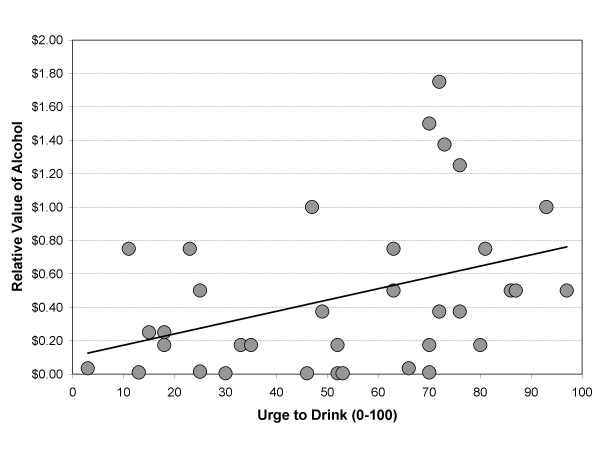
Continuous association between urge (i.e., craving) for alcohol and the relative value of alcohol from an alcohol-money choice task; β = .38, *p *< .05.

Following the factorial analyses (i.e., induction condition), continuous analyses were conducted between the experiential variables (i.e., craving and affect) and the relative value of alcohol using the same regression-based approach, but with post-induction variable values serving as the independent variable. Continuous examination of the relationship between post-cue exposure craving and the relative value of alcohol revealed a significant association (*R*^2 ^= .15, *F *(1, 33) = 5.711, β = .38, *p *< .05), reflecting a positive relationship between craving and relative value of alcohol, as depicted in Figure 2. Neither positive (*R*^2 ^= .00, *F *(1, 33) = .00, *p *> .90), nor negative affect (*R*^2 ^= .00, *F *(1, 33) = .00, *p *> .90) were significantly associated with relative value of alcohol.

### Influences of DRD4 VNTR genotype

To examine the influence of DRD4 VNTR genotype, DRD4 VNTR status and the induction-by-genotype interaction term were added to the preceding hierarchical multiple regression analyses of induction effects. Specifically, the post-induction value of a variable served as the dependent variable, its pre-induction level was entered as a covariate in a first block (with the exception of relative value of alcohol, which had no baseline level), induction type was entered in a second block, and genotype and the interaction term were entered in a third block; DRD4 VNTR status was coded as DRD4-S = 1 and DRD4-L = 2. Frequencies of genotypes revealed the following cell sample sizes for the factorial analyses: craving induction-DRD4-S = 10; craving induction-DRD4-L = 8; control induction-DRD4-S = 14; control induction-DRD4-L = 3. Given the preceding cell sizes, particularly the latter, the 2 × 2 factorial analyses had very limited power but were nonetheless conducted. In terms of craving, the addition of genotype and induction-by-genotype interaction term did not significantly improve the model (Δ*R*^2 ^= .01, *F *(2, 30) = .39, *p *> .65), as was the case for relative value of alcohol (Δ*R*^2 ^= .08, *F *(2, 31) = 1.24, *p *> .40), positive affect (Δ*R*^2 ^= .00, *F *(2, 30) = .10, *p *> .80) or negative affect (*F *(2, 29) = .09, *p *> .80).

**Figure 3:**
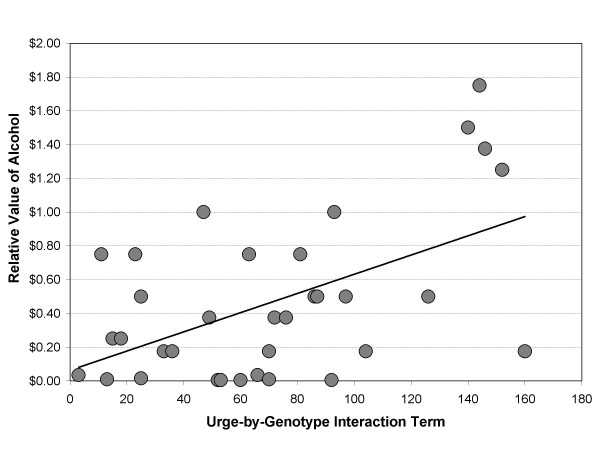
Association between the craving by DRD4 VNTR genotype interaction term and the relative value of alcohol (β = 1.70, *p *< .01).

The issue of small cell sample size did not apply to the continuous analyses and, in the case of craving, the addition of DRD4 VNTR status and the interaction term resulted in a significant improvement of the model (Δ*R*^2 ^= .22, F (1, 33) = 5.22, *p *= .01). Variable coefficients are provided in Table 2 and revealed that this improvement was accounted for by a significant association for the craving-by-genotype interaction. The interaction effect is depicted in Figure 3 and reflected a relationship such that the influence of craving on the relative value of alcohol was disproportionately high for DRD4-L individuals. No significant independent effects or moderating effects of DRD4 VNTR status were evident for positive (Δ*R*^2 ^= .13, *F *(2, 31) = 2.27, *p *> .10) or negative affect (Δ*R*^2 ^= .09, *F *(2, 31) = 1.50, *p *> .20).

**Table 2:** Associations between craving, DRD4 VNTR genotype, and the interaction with the relative value of alcohol.

Variable	*B*	*SE B*	β	*R*^2^
*Craving Model*				0.15
Craving	0.007	0.003	.38*	
*Combined Model*				0.36
Craving	-0.015	0.008	-0.86	
DRD4 VNTR Genotype	-0.767	0.387	-0.77	
Craving × Genotype Interaction	0.018	0.007	1.70**	

Regression coefficient estimates for the craving model including only craving, and for the combined model including craving, DRD4 VNTR genotype, and the interaction term with the relative value of alcohol as the dependent variable. Unstandardized (± SE) and standardized beta weights are provided, with the total variance accounted for by the model (*R*^2^) and statistical significance; * *p *< .05, ** *p *< .01.

### Population stratification

The likelihood of population stratification was considered low because there is no evidence to date of population stratification for the phenotypes under consideration (i.e., craving, relative value of alcohol, affect). In addition, the overall sample in this study was relatively small and the large majority of participants were of the same race (Caucasian); proportions of participants by genotype and by race are shown in Table 3. Nonetheless, to address the possibility of population stratification, the significant genetic findings were re-conducted using only the Caucasian participants, which revealed the same pattern of findings as the principal analyses. The preceding combined model (i.e., craving, genotype, craving-by-genotype interaction) was significant (*p *< .001), neither craving, nor DRD4 VNTR coefficients were independently significant (*p*s > .30), and the interaction coefficient was significant (*p *< .05), reflecting the same positive relationship. Formal tests of population stratification using genomic control were not conducted.

**Table 3:** Genotype by race/ethnicity.

	Caucasian	Hispanic	Asian
DRD4-S (*n *= 24)	79%	17%	4%
DRD4-L (*n *= 11)	91%	9%	0%

## Discussion

This preliminary examination of the influences of craving for alcohol and DRD4 VNTR genotype on the relative value of alcohol generated mixed results. As anticipated, the craving induction resulted in a significant increase in subjective craving compared to the control induction, but contrary to predictions, it was not associated with greater relative value of alcohol. However, when the data were considered continuously, independent of experimental condition, craving was significantly associated with the relative value of alcohol. Parallel findings were evident when considering the influence of DRD4 VNTR genotype. DRD4-L status did not significantly moderate the effects of the alcohol cue exposure, but when considered continuously, DRD4 VNTR genotype significantly moderated the relationship between craving and the relative value of alcohol. The form of this relationship was such that as craving increased, DRD4-L individuals exhibited a disproportionately greater relative value of alcohol. Across the procedures, affect was not influenced by the craving induction and was not associated with the relative value of alcohol. To most clearly discuss these findings, we will first consider the specific findings relating to the craving induction and will then consider the role of DRD4 VNTR genotype.

### Effects of the craving induction

With regard to the effects of the craving induction compared to the control induction, the discrepancy between the factorial and continuous findings provides only mixed support for the visceral theory. On one hand, the lack of effect of the alcohol cue exposure is in direct contrast to what the visceral theory would predict, but, on the other hand, the significant continuous association between craving and relative value is consistent with the visceral theory's prediction that the absolute level of craving determines the relative value of a commodity. Although these findings appear to be contradictory, they may be understood in the context of the cue exposure craving induction itself. Although the cue exposure paradigm typically generates significant increases in craving in aggregate, there is considerable variation in participants' reactions and in the absolute levels of craving that are reported [48,49]. This is compounded by the fact that participants report a wide array of levels of craving in general prior to the procedure, which contributes to the overall heterogeneity. For example, in the current study, craving at baseline ranged from 3 to 79 out of 100. Thus, although subjects receiving the alcohol cue exposure may have reported greater craving in aggregate, it appears that was still sufficient heterogeneity between the two groups for that effect to not translate into a significant effect on relative value.

In addition, some additional methodological considerations may be relevant to no significant effect of the craving induction on the relative value of alcohol. First, although the relative value task was intentionally designed to be short and provide immediate tangible rewards, it could only capture one facet of relative value, which is itself a multidimensional construct [70,71]. Second, the relative value of alcohol task only used a maximum of one standard alcoholic beverage, which would have relatively limited psychoactive effects and may have affected participant's valuation of the beverage. Third, the task was administered on only one occasion because it directly provided alcohol which would have affected subsequent performance. However, as a result, it could not be administered on multiple occasions and thus did not provide a baseline relative value of alcohol. Each of these aspects of the methodology could have contributed to the lack of effect in the factorial analyses and should be considered in future studies.

Despite these potential explanations for the factorial findings, performance on the relative value of alcohol task was nonetheless sensitive to variation in craving in the continuous analyses, which accords with the prediction of the visceral theory. Indeed, a cornerstone of the theory is that the absolute level of craving is a critical determinant of behaviour, which is also supported by the current data where overall experiential craving was more important than the experimental manipulation.

### Influences of DRD4 VNTR genotype

Like the effects of the craving induction, the influence of DRD4 VNTR genotype was mixed, exhibiting a negligible influence in the factorial analyses but a substantial influence in the continuous analyses. Although potential methodological explanations for this discrepancy are the same as those described in the preceding section, it is also important to note that the study was relatively small in general and retrospective genotyping resulted in very few subjects in one cell in the induction-by-genotype factorial analyses. As a result, the factorial genetic analyses had very low statistical power, substantially limiting the interpretability of those findings. Importantly, rather than interpreting the lack of moderating effect of DRD4 VNTR genotype on reaction to alcohol cues as diverging from previous findings [37,40], it appears more prudent to conclude that this study could not fully test that hypothesis.

In contrast, in the continuous analyses where power was not limited by cell size, the data conformed to predictions. As self-reported craving increased, those individuals possessing the long variant exhibited greater valuation of alcohol on the behavioral task. These data are broadly consistent with previous studies that have suggested that DRD4-L status is associated with amplified expression of craving for alcohol [37-40], as well as other appetitive targets, such as cigarettes [45], heroin [46], and food [47]. Of note, although the threat of population stratification is low in general [69] and was considered to be low in this study, this was confirmed by replicating the principal findings in the subsample reporting Caucasian ancestry.

From a mechanistic standpoint, these findings are consistent with Hutchison et al.'s [45] proposal that possession of a DRD4-L allelic variant enhances an individual's sensitivity to dopaminergic rewards, including alcohol use. Although characterizing the functional significance of polymorphisms of the DRD4 VNTR genotype remains an active basic research question [72], Hutchison et al. [45] propose that this influence may be via functional differences between the polymorphisms in accumulation of cyclic adenosine monophosphate (cAMP) in response to dopamine agonism. D_4 _receptors are members of the D_2 _receptor family and, as such, inhibit cAMP formation, however, the long DRD4 VNTR variant permits two to three times greater accumulation of cAMP [32]. As a result, DRD4-L individuals may be inferred to have chronically elevated levels of cAMP, which, in conjunction with elevations of additional downstream transcriptional factors, has been associated with enhanced sensitivity to dopaminergic rewards in a number of studies using animal models [73-75]. Moreover, such effects are specific to increased in cAMP in the nucleus accumbens, which is both a structure where D_4 _receptors are localized [76] and one of the key putative substrates of reward motivation [34,35]. However, despite the plausibility of differences in intracellular cAMP being responsible for the observed interaction, because the functional role of polymorphisms of the DRD4 VNTR genotype has not been fully characterized [72] and the literature on DRD4 VNTR influences on craving in humans is relatively small, it is important to underscore that considerable further research is necessary to understand these relationships.

### Future directions

Based on this preliminary study, further examinations of the utility of the visceral theory for understanding craving as a motivational factor appear to be warranted. Methodologically, as noted above, subsequent studies would do well to consider behavioural economic methodologies that allow for dynamic point-to-point assessments of relative value that can incorporate multiple facets of value and potentially more meaningful amounts of alcohol. Clearly, larger sample sizes would permit more exhaustive examinations of genetic contributions to craving, both in terms of statistical power and the alleles under consideration. DRD4 VNTR was the single target of the current study based on the existing literature [37,38,40] and the small sample size, however, other recent studies have suggested provocative interactions with other alleles. These include positive moderating effects of craving inductions by minor alleles of the OPRM1, DRD2 *TaqI *A, and SLC6A3 genes [[77,78]; cf. [37]], although at this point these represent isolated reports. Finally, it is possible that measures of relative value would have utility in neuroimaging research characterizing the neurobiological structures that subserve addictive behaviour. An extensive literature has demonstrated that craving inductions selectively activate the anterior cingulate, dorsolateral prefrontal cortex, and orbitofrontal cortex [79-84], however, less is known about which of these structures directly influence subsequent choices for a substance. Such an application would be an archetypal example of the newly burgeoning transdisciplinary field of neuroeconomics [85].

## Conclusion

This study used Loewenstein's visceral theory of addiction as the basis for examining the influences of a craving induction and DRD4 VNTR genotype on the relative value of alcohol. Factorial analyses revealed a significant effect of the craving induction on craving, but not on the relative value of alcohol and no interaction with DRD4 VNTR genotype. In contrast, continuous analyses revealed the predicted relationships, with craving significantly associated with the relative value of alcohol and DRD4 VNTR moderating the strength of this relationship. Taken together, these findings are interpreted as generally supporting the potential utility of the visceral theory and further demonstrating the relevance of DRD4 VNTR genotype to understanding the expression of craving for alcohol.

## Competing interests

The author(s) declare that they have no competing interests.

## Authors' contributions

JM conceived the study, developed the procedures, analyzed the data and was the principal author of the manuscript. DM contributed to the conception of the study, ran all participants, and contributed to data interpretation and manuscript preparation. JMc genotyped all participants, contributed to data interpretation, and participated in manuscript preparation. SAL contributed to the conception of the study, data interpretation, and manuscript preparation. All authors have read and approved this manuscript.
